# Transcriptomic Profiling Reveals Intense Host-Pathogen Dispute Compromising Homeostasis during Acute Rift Valley Fever Virus Infection

**DOI:** 10.1128/jvi.00415-23

**Published:** 2023-06-12

**Authors:** Erick Bermúdez-Méndez, Paolo Angelino, Lucien van Keulen, Sandra van de Water, Barry Rockx, Gorben P. Pijlman, Angela Ciuffi, Jeroen Kortekaas, Paul J. Wichgers Schreur

**Affiliations:** a Department of Virology and Molecular Biology, Wageningen Bioveterinary Research, Lelystad, The Netherlands; b Laboratory of Virology, Wageningen University & Research, Wageningen, The Netherlands; c Institute of Microbiology, Lausanne University Hospital, Lausanne, Switzerland; d Institute of Microbiology, University of Lausanne, Lausanne, Switzerland; e Bioinformatics Core Facility, Swiss Institute of Bioinformatics, Lausanne, Switzerland; f Department of Bacteriology, Host-Pathogen Interaction and Diagnostics Development, Wageningen Bioveterinary Research, Lelystad, The Netherlands; University of Kentucky College of Medicine

**Keywords:** host-pathogen interactions, pathogenesis, RNA-seq, Rift Valley fever virus

## Abstract

Rift Valley fever virus (RVFV) (family *Phenuiviridae*) can cause severe disease, and outbreaks of this mosquito-borne pathogen pose a significant threat to public and animal health. Yet many molecular aspects of RVFV pathogenesis remain incompletely understood. Natural RVFV infections are acute, characterized by a rapid onset of peak viremia during the first days post-infection, followed by a rapid decline. Although *in vitro* studies identified a major role of interferon (IFN) responses in counteracting the infection, a comprehensive overview of the specific host factors that play a role in RVFV pathogenesis *in vivo* is still lacking. Here, the host *in vivo* transcriptional profiles in the liver and spleen tissues of lambs exposed to RVFV are studied using RNA sequencing (RNA-seq) technology. We validate that IFN-mediated pathways are robustly activated in response to infection. We also link the observed hepatocellular necrosis with severely compromised organ function, which is reflected as a marked downregulation of multiple metabolic enzymes essential for homeostasis. Furthermore, we associate the elevated basal expression of *LRP1* in the liver with RVFV tissue tropism. Collectively, the results of this study deepen the knowledge of the *in vivo* host response during RVFV infection and reveal new insights into the gene regulation networks underlying pathogenesis in a natural host.

**IMPORTANCE** Rift Valley fever virus (RVFV) is a mosquito-transmitted pathogen capable of causing severe disease in animals and humans. Outbreaks of RVFV pose a significant threat to public health and can result in substantial economic losses. Little is known about the molecular basis of RVFV pathogenesis *in vivo*, particularly in its natural hosts. We employed RNA-seq technology to investigate genome-wide host responses in the liver and spleen of lambs during acute RVFV infection. We show that RVFV infection drastically decreases the expression of metabolic enzymes, which impairs normal liver function. Moreover, we highlight that basal expression levels of the host factor *LRP1* may be a determinant of RVFV tissue tropism. This study links the typical pathological phenotype induced by RVFV infection with tissue-specific gene expression profiles, thereby improving our understanding of RVFV pathogenesis.

## INTRODUCTION

Rift Valley fever virus (RVFV) (family *Phenuiviridae*) is a single-stranded, three-segmented, negative-sense RNA virus transmitted by mosquitoes (1–3). RVFV mainly affects ruminants such as sheep, goats, and cattle but can also affect camelids and humans (4–6). Infected animals generally present with fever, anorexia, diarrhea, and overall weakness. Epizootic outbreaks are commonly characterized by abortion storms in sheep flocks and high mortality rates among newborns (1, 7). In humans, the clinical presentation of the disease is characterized by symptoms such as fever, headache, nausea, vomiting, abdominal pain, and diarrhea. In a minority of cases, the infection may progress to severe disease leading to hepato-renal failure, encephalitis, retinitis, and/or hemorrhagic manifestations (8–10).

In both animals and humans, the liver is the primary site of RVFV replication. Histopathological examinations of infected liver tissues revealed that diseased animals exhibit multifocal lesions and necrotic hepatitis (11, 12). The spleen is also commonly targeted during RVFV infection, with microscopic examination revealing various degrees of necrosis. Apart from the liver and the spleen, RVFV can occasionally be found in the kidneys, lungs, skin, brain, and placenta (11, 13–15).

RVFV is endemic to most countries on the African Continent and the Arabian Peninsula. Nevertheless, as competent mosquito vectors already inhabit other geographical regions and are still expanding their territory, facilitated by climate change and international trade and transport, it is likely that the virus will be capable of invading previously unaffected areas (16–19). RVFV outbreaks represent a significant threat to public health and can result in substantial economic losses (4, 20, 21), yet many key aspects of RVFV infection cycle and the molecular mechanisms underlying its pathogenesis are poorly comprehended.

High viremia peaking at 2 to 3 days post-infection followed by an abrupt decline from 4 to 5 days post-infection onward is a signature feature of acute RVFV infections in ruminants (22). Generally, infected animals either succumb or fully recover during these days. Previous studies investigating RVFV virulence factors and pathogenesis revealed the predominant role of the non-structural protein NSs as an innate immune response antagonist. NSs acts primarily through the blockage of type I interferon (IFN) (IFN-α/β) production by inhibiting host cell transcription (23–25) and by inducing the degradation of the double-stranded RNA-dependent protein kinase (PKR) (26–28).

Recently, RNA sequencing (RNA-seq) technologies have enabled the transcriptome-wide analysis of host responses during virus infections. RNA-seq studies benefit from an exhaustive approach to investigate the expression of the whole transcriptome using high-throughput sequencing instead of focusing on a short list of predicted genes of interest (29, 30). This broad analysis allows the detection of novel host factors playing an important role in the infection cycle that have escaped the radar of single-pathway-oriented investigations.

Two previous RVFV-mammalian host RNA-seq studies have been carried out with samples derived from infected cell cultures. The first study investigated the cellular response upon infection with the attenuated MP-12 strain and the virulent ZH548 strain in human small airway epithelial cells. In that study, the top pathways altered in response to infection with both RVFV strains included the regulation of the antiviral response, mitochondrial dysfunction, the DNA damage response, and integrin-linked kinase (ILK) signaling (31). The second study investigated the response induced by MP-12 in HEK293 human embryonic kidney cells. The activation of innate immune signaling pathways and the upregulation of pro-inflammatory cytokines were observed, as were alterations in pathways associated with fatty acid metabolism and extracellular matrix receptor signaling (32).

Although these *in vitro* studies confirmed that IFN-mediated responses are crucial for fighting RVFV infection, our understanding of the tissue-specific host factors that play a role in *in vivo* pathogenesis is still very limited. To date, only one *in vivo* transcriptomic study on a RVFV-infected mammalian (surrogate) host has been performed. Investigation of the immune response in the brain of mice intranasally infected with RVFV revealed a protective response mediated by the mitochondrial antiviral-signaling protein (MAVS) (33). A better understanding of the virus-host interface, especially in a natural RVFV host, would benefit the development of more effective outbreak control strategies.

In this work, we used RNA-seq technology to study *in vivo* the host transcriptional profiles in the liver and spleen tissues of lambs during RVFV peak viremia. We compared the gene expression profiles of infected lambs at 2 and 4 days post-infection with those of uninfected lambs and performed a genome-wide pathway analysis to identify biological processes that are affected during an acute infection. We revealed gene expression signatures underlying the observed histopathology phenotype, in addition to confirming the transcriptional responses to RVFV previously identified by *in vitro* experiments. Thus, this study improves our understanding of the *in vivo* host response to RVFV infection and uncovers new molecular features of RVFV pathogenesis.

## RESULTS

### Selection of RVFV-infected ovine tissue samples for transcriptome analysis.

**FIG 1:**
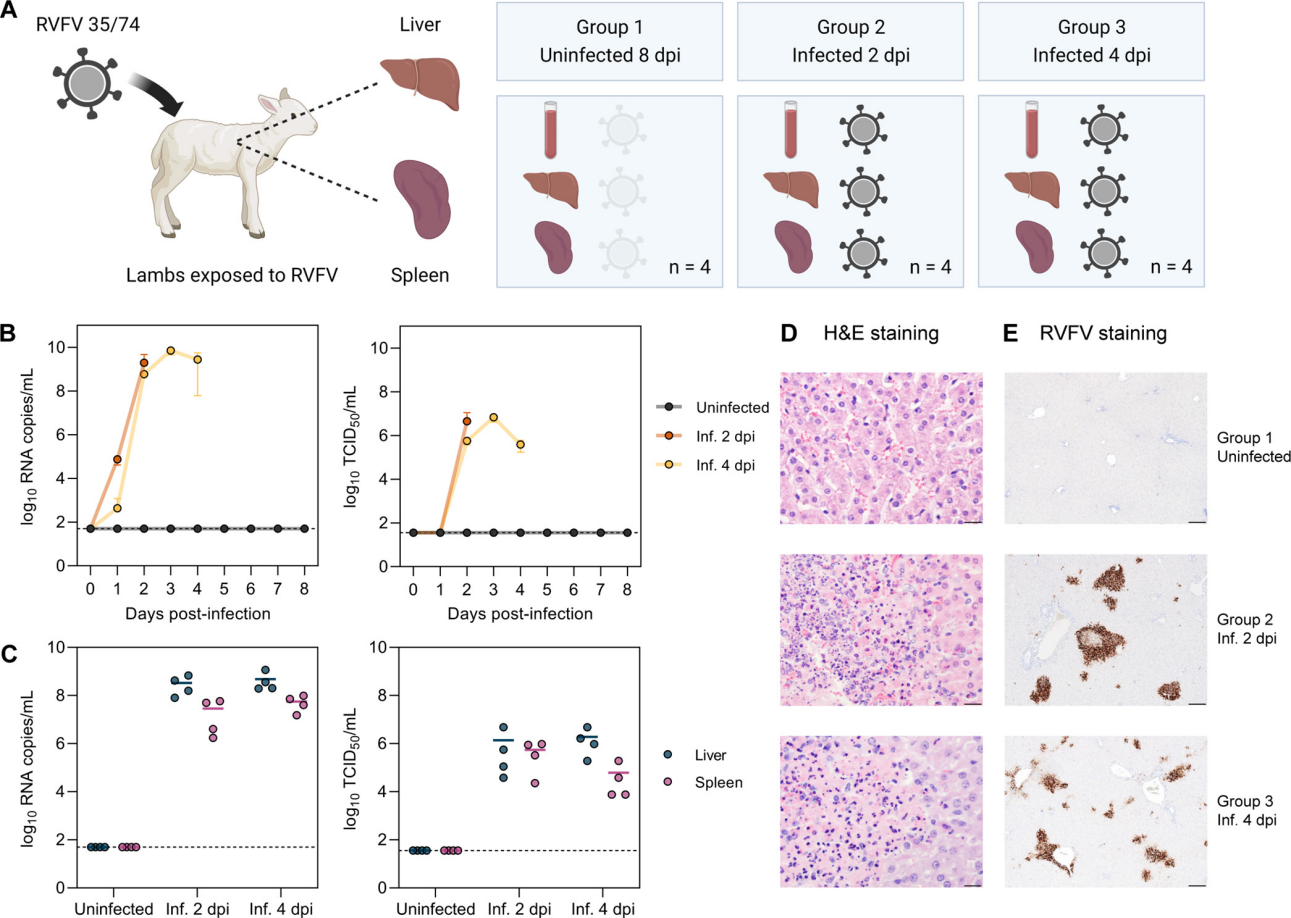
Selection of biologically relevant RVFV-infected samples. (A) Schematic representation of the selected animal samples. Liver and spleen samples of lambs exposed to RVFV strain 35/74 were selected from another study (34). Group 1 consists of uninfected (non-responsive) lambs necropsied at 8 days post-exposure. Group 2 and group 3 consist of infected lambs necropsied at 2 and 4 days post-infection (dpi), respectively (*n* = 4 samples per group). (B and C) Viral RNA copy numbers and infectious titers in the blood (B) and target organs (C). Viral RNA was quantified by M-segment-specific RT-qPCR, and infectious titers were determined by an endpoint dilution virus isolation assay (34). In panel B, graphs show the means with standard deviations (SD) at each time point. In panel C, dots represent individual replicates, and the horizontal lines represent the means. Dashed lines indicate the limits of detection (50 RNA copies/mL for RT-qPCR and 35.5 median tissue culture infectious doses [TCID_50_]/mL for the virus isolation assay). (D) Hematoxylin and eosin (H&E) staining of liver tissue sections. Group 1 shows no histological alterations in the liver, while groups 2 and 3 show acute hepatitis with necrosis of hepatocytes and an influx of polymorphonuclear cells. Bars, 20 μm. (E) Immunohistochemical detection of RVFV antigen in liver tissue. RVFV Gn glycoprotein (brown) was detected with antibody 4-D4 (61) in combination with HRP-conjugated secondary antibodies. Bars, 200 μm. Inf., infected.

Since sheep are the primary natural hosts of RVFV, and the liver and spleen are the two main target organs of the virus, we selected liver and spleen samples from lambs with moderate to high levels of viral RNA for host transcriptome analysis. For comparison, we selected samples from uninfected lambs. All the tissue samples were obtained from an experiment with Texel-Swifter lambs that were exposed to RVFV either via intravenous injection or via bites from infected mosquitoes (34) (Fig. 1A; see also Materials and Methods for additional details). Samples from group 1 belonged to lambs that were exposed to a low number of infected mosquitoes. These lambs did not develop any signs of disease, nor did they show detectable levels of infectious virus or viral RNA in the blood or the target organs (Fig. 1B and C). Group 1 was thus considered the control group. Samples from group 2 and group 3 belonged to lambs necropsied on days 2 and 4 post-infection, respectively, that presented with high levels of viral RNA (ranging between 10^7^ and 10^10^ copies/mL on average) and infectious virus (ranging between 10^4^ and 10^7^ median tissue culture infectious doses [TCID_50_]/mL on average) in the blood and the target organs (Fig. 1B and C). Noteworthy, the RNA copy numbers and infectious titers were slightly higher in the liver samples than in the spleen samples, without appreciable differences between lambs of groups 2 and 3.

### Histological examination confirmed the infection status.

Upon histological examination of tissue sections of the liver, acute necrosis of hepatocytes and an influx of neutrophils were observed in lambs from groups 2 and 3, clear indicators of acute hepatitis. In contrast, no histological alterations were observed in the liver tissues of the control lambs (group 1) (Fig. 1D). In line with the histological observations, immunohistochemistry detection of RVFV antigen revealed foci of infected cells in the livers of lambs from groups 2 and 3. The majority of the infected cells corresponded to necrotic hepatocytes, and a minority corresponded to endothelial cells. No virus-specific staining was observed in samples from group 1 (Fig. 1E).

### RVFV infection status determines characteristic gene expression profiles.

To determine the genome-wide gene expression profile in response to RVFV infection in the liver and spleen, total RNA from frozen tissue samples preserved in RNA*later* was isolated and subjected to poly(A)-enriched RNA-seq-based host transcriptome analysis. The integrity of the isolated RNA was confirmed by assessment of the RNA quality number (RQN) or the RNA integrity number (RIN) (35), with RQN or RIN values >6 being found for the majority of the samples (see Table S1 in the supplemental material). At least 20 million high-quality reads (average Phred score >30) per sample were obtained and mapped (ranging from 61.1% to 82.4% alignment) to the sheep (Ovis aries) NCBI reference genome (Fig. S1A to C). A summary of the next-generation sequencing run and general statistics are provided in Table S2. Initially, a total of 26,200 genes were detected across the different samples, of which 18,005 genes were retained for analysis after applying a filter to keep only genes with counts of 3 or higher in at least 2 of the samples. Of the input genes retained for the analysis, 83.8% were annotated in the reference genome (Fig. S1D).

**FIG 2:**
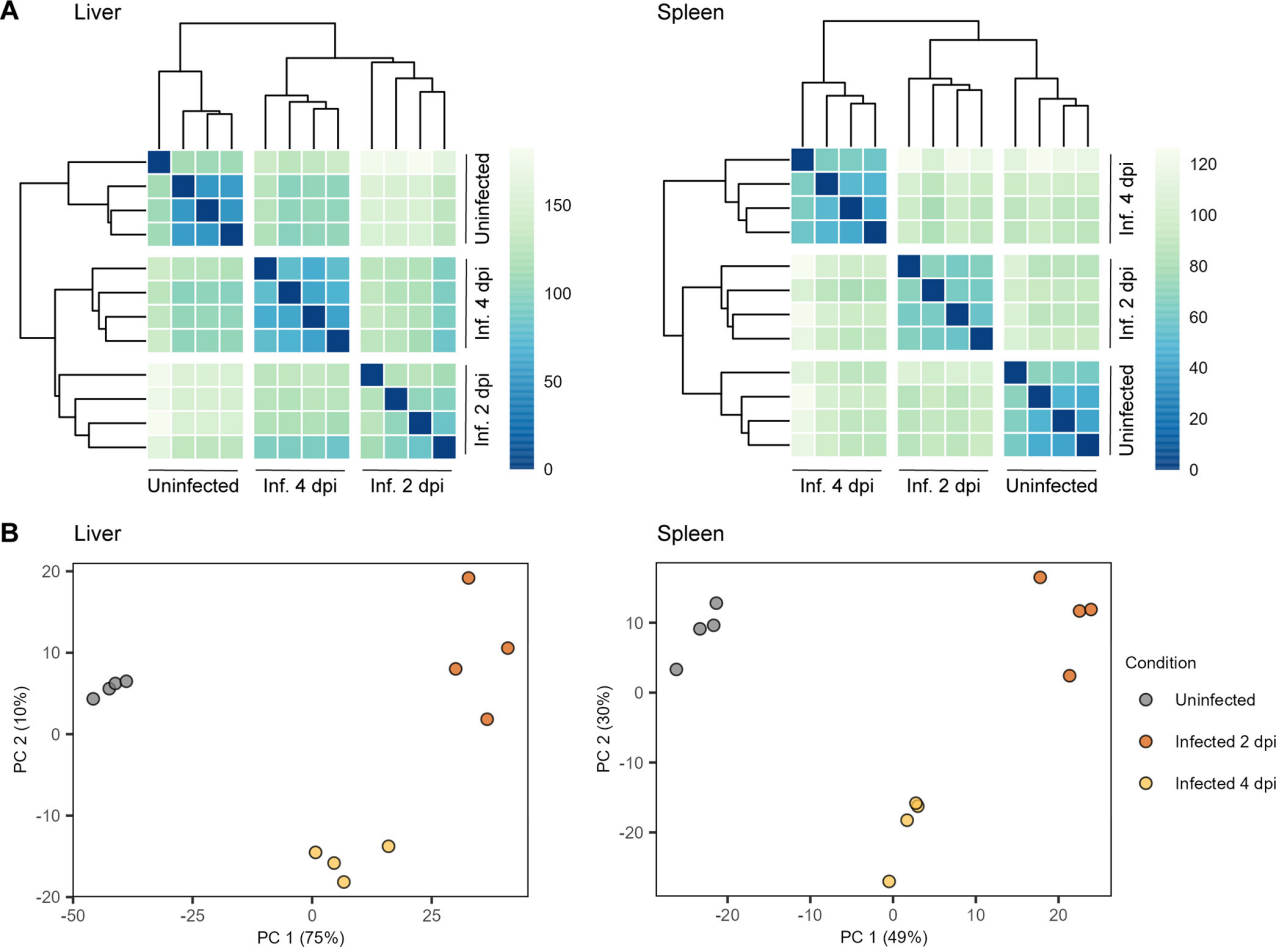
RVFV infection status determines characteristic gene expression profiles. (A) Heat maps of the Euclidean distances between liver (left) and spleen (right) tissue samples. Based on the calculated distance, each cell is color-coded in shades of a sequential gradient ranging from dark blue (close distance, implying similarity) to light green (far distance, implying dissimilarity). (B) Principal component analyses of liver (left) and spleen (right) tissue samples. Within each tissue type, samples cluster into three distinct well-defined clusters corresponding to a particular infection status. Abbreviations: Inf., infected; dpi, days post-infection.

Separation mostly between liver and spleen samples was observed by both heat maps of the Euclidean distances and principal component analysis when all the samples were analyzed together (Fig. S2A and B). As expected, this separation indicates that the gene expression profiles depend first on the host tissue rather than the infection status. We next performed similar analyses of each tissue separately. Heat maps of the Euclidean distances between samples revealed groupings into three distinct clusters (Fig. 2A). This clustering was also evident in the principal component analysis, where PC1 and PC2 represented 75% and 10% of the variance in the liver samples and 49% and 30% of the variance in the spleen samples, respectively (Fig. 2B). Notably, each cluster corresponded to either the uninfected lambs (group 1), the lambs at 2 days post-infection (group 2), or the lambs at 4 days post-infection (group 3), indicating that the infection status of the lamb determines a characteristic gene expression profile in each tissue.

### RVFV infection induces extensive changes in liver and spleen gene expression.

**FIG 3:**
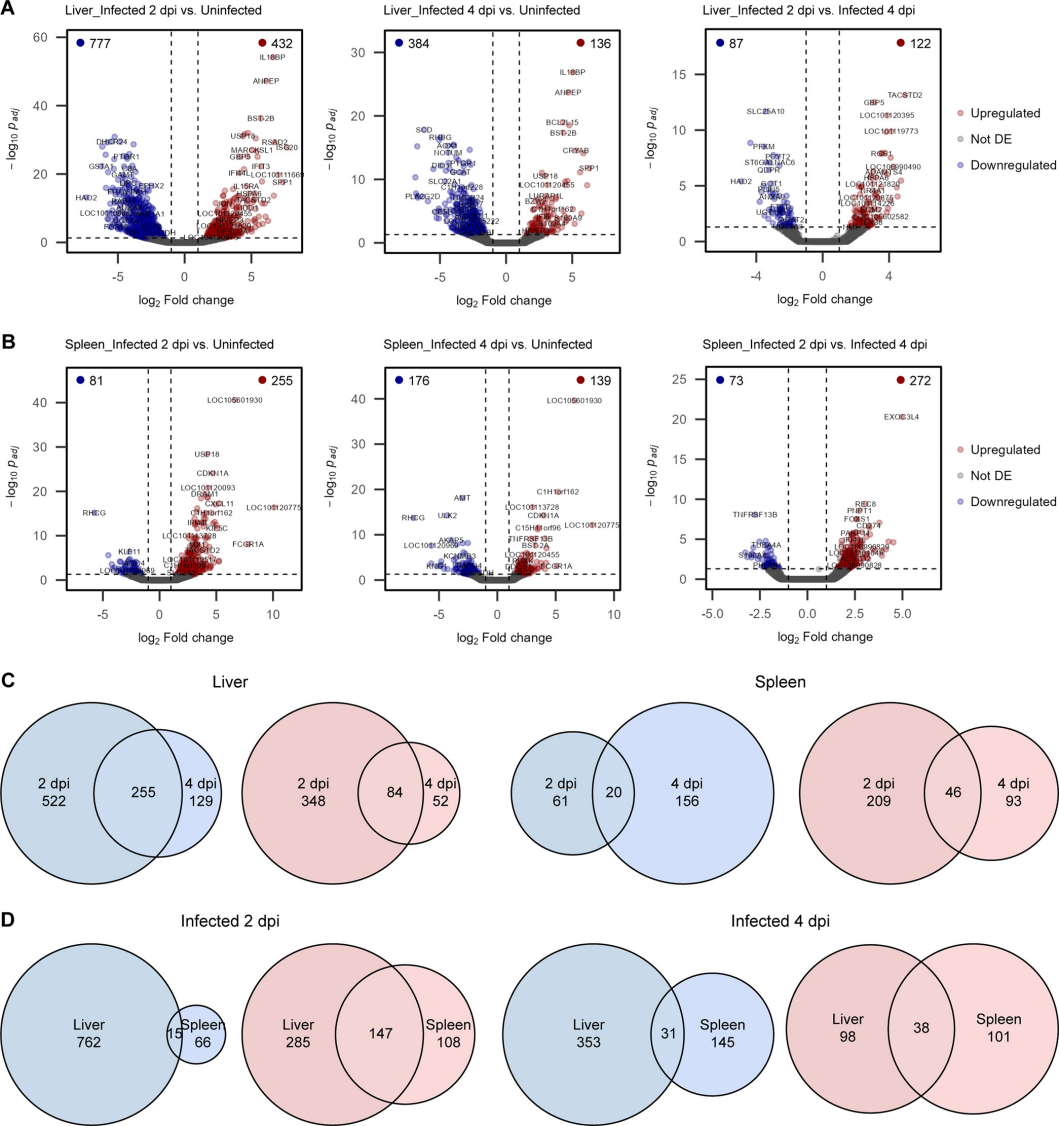
RVFV-induced changes in liver and spleen gene expression. (A and B) Volcano plots of differential gene expression analysis in liver (A) and spleen (B) tissues. Uninfected (group 1) and infected (groups 2 and 3) samples were compared pairwise. Dots represent individual genes. Genes with an absolute log_2_ fold change of 1 and a Wald test *P* value (adjusted by the Benjamini-Hochberg method) <0.05 were considered significantly differentially expressed (DE). The vertical and horizontal dashed lines indicate the log_2_ fold changes and adjusted *P* value thresholds, respectively. The numbers of significantly downregulated (blue) and upregulated (red) genes are indicated at the top corners of each plot. (C and D) Euler diagrams representing the numbers of shared (intersection) and unique downregulated (blue) and upregulated (red) genes between infected groups 2 and 3 (compared to control group 1). Comparisons of time points for the same tissue type (C) and comparisons of tissues at the same time point (D) are shown. Abbreviation: dpi, days post-infection.

To examine how the infection status influenced the host liver and spleen responses upon RVFV infection, we performed pairwise differential gene expression analysis between the samples of each tissue type. Genes with an absolute log_2_ fold change of 1 and an adjusted *P* value <0.05 were considered significantly differentially expressed. In all of the paired comparisons, tens to hundreds of genes were differentially expressed (down- or upregulated), confirming that RVFV infection induced significant changes in the host liver and spleen transcriptomes (Fig. 3A and B). Lists of all of the differentially expressed genes with their corresponding log_2_ fold changes and adjusted *P* values are provided in Table S3.

### RVFV infection leads to immune response activation and decreased tissue-specific function.

Within each tissue type, a fraction of differentially expressed genes were common to infected samples (groups 2 and 3) compared to uninfected samples (group 1), as depicted by the genes belonging to the intersection (overlap) of the corresponding gene lists (Fig. 3C and Table S4). This commonality between the infected groups indicates that some genes remain either down- or upregulated during the peak phase (day 2) and the start of virus clearance (day 4) of an acute infection. Interestingly, when comparing samples between tissue types but at the same time points, there was a substantial intersection of genes upregulated in both the liver and the spleen. This intersection of upregulated genes in both tissues is mainly due to genes involved in the host’s immune response against viral infection. On the contrary, the intersection of downregulated genes between both tissue types was minor, suggesting that downregulated gene expression changes are tissue specific (Fig. 3D and Table S4).

**FIG 4:**
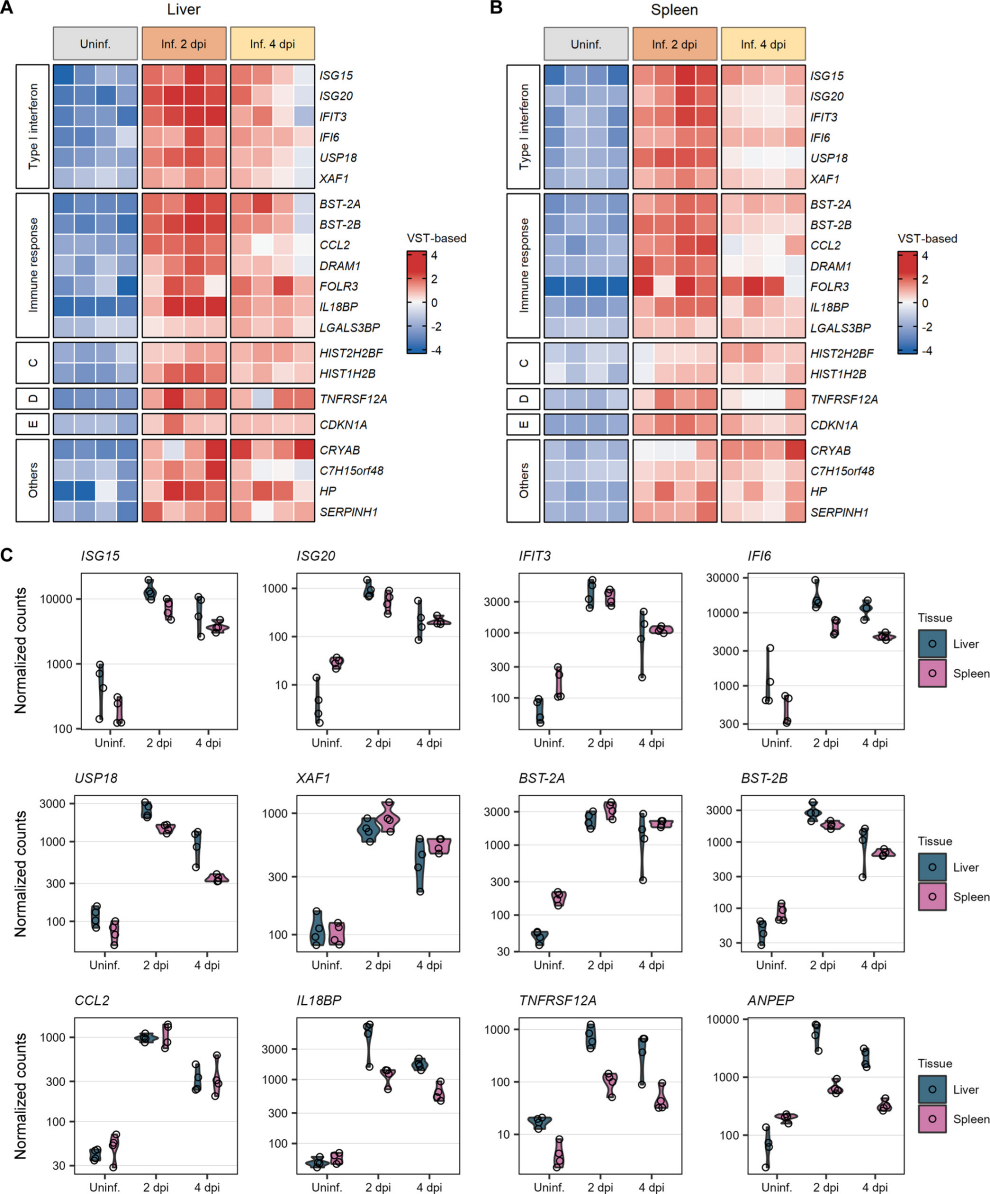
Commonly upregulated genes in the liver and spleen during peak RVFV infection. (A and B) Heat maps of a set of 21 genes commonly upregulated after RVFV infection in liver (A) and spleen (B) tissues. Samples were clustered based on their infection status, as indicated on top of the heat maps. Genes were clustered into categories based on their molecular function or biological process, as indicated at the left of the heat maps. To represent the magnitude of the log_2_ fold change of each gene compared to the mean gene expression level, cells are color-coded in shades of a gradient ranging from dark blue (low) to dark red (high). (C) Expression profiles of 12 selected genes commonly upregulated in RVFV-infected (Inf.) samples at 2 days post-infection (dpi) (group 2) and 4 days post-infection (group 3) compared to uninfected (Uninf.) samples (group 1). Dots represent individual normalized gene counts (on a log_10_ scale), and the shaded area shows the distribution of the samples within each group. Abbreviations: VST, variance-stabilizing transformation; C, nucleosome assembly; D, cytokine receptor; E, apoptosis; Others, a chaperone and genes involved in metabolic processes such as collagen biosynthesis.

A selection of the top 50 most variably expressed genes in the liver and spleen not only separated the different groups of samples based on infection status but also showed divergent patterns of expression in specific subsets of genes (Fig. S3). In both liver and spleen tissues, the largest differences were observed between the uninfected (group 1) and the 2 days post-infection (group 2) samples. Remarkably, some of the most variably expressed genes showing the largest divergences in expression patterns belonged to a set of 21 genes that were upregulated in the liver and spleen across all the infected samples (2 and 4 days post-infection) (Fig. 4A to C). Most of these genes, including *ISG15*, *ISG20*, *IFIT3*, *IFI6*, *USP18*, *XAF1*, *BST-2A*, *BST-2B*, *CCL2*, *DRAM1*, *FOLR3*, *IL18BP*, and *LGALS3BP*, are involved in the immune response of the host against viral infection, mainly via IFN-mediated signaling pathways. The remaining genes are involved in, among others, nucleosome assembly, apoptosis, and collagen biosynthesis.

**FIG 5:**
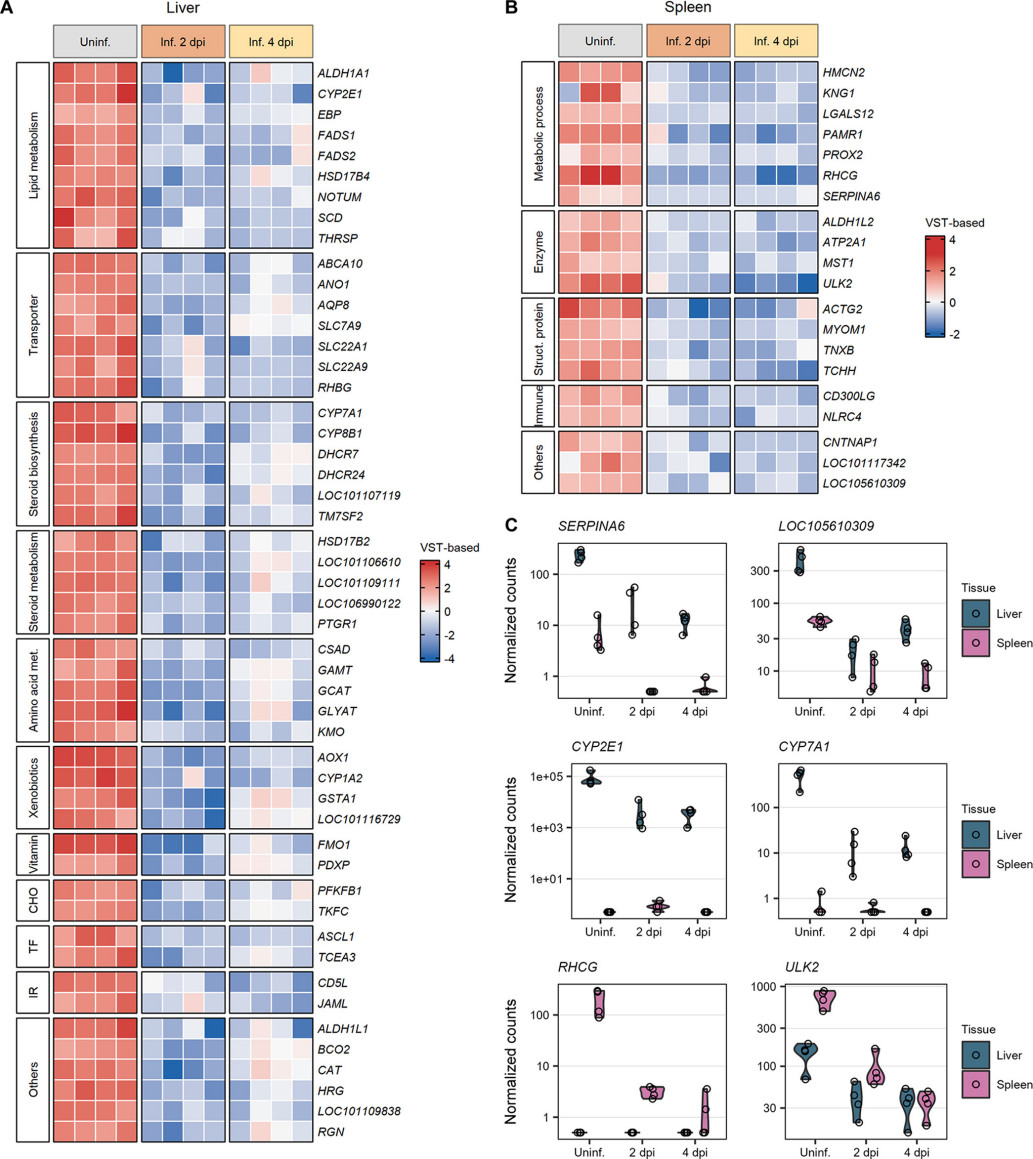
Downregulated genes in the liver and spleen during peak RVFV infection. (A and B) Heat maps of sets of genes significantly downregulated after RVFV infection in liver (A) and spleen (B) tissues. Samples were clustered based on their infection status, as indicated on top of the heat maps. Genes were clustered into categories based on their molecular function or biological process, as indicated at the left of the heat maps. To represent the magnitude of the log_2_ fold change of each gene compared to the mean gene expression level, cells are color-coded in shades of a gradient ranging from dark blue (low) to dark red (high). (C) Expression profiles of 6 selected genes significantly downregulated in RVFV-infected (Inf.) liver or spleen samples at 2 days post-infection (dpi) (group 2) and 4 days post-infection (group 3) compared to uninfected (Uninf.) samples (group 1). Dots represent individual normalized gene counts (on a log_10_ scale), and the shaded area shows the distribution of the samples within each group. Abbreviations: VST, variance-stabilizing transformation; met., metabolism; CHO, carbohydrate metabolism; TF, transcription factor; IR, immune response; Struc., structural; Others, genes involved in folate metabolism, carotenoid metabolism, purine metabolism, signaling receptor activity, cell growth, hemostasis, and senescence.

As initially noticed in the Euler diagram (Fig. 3D), the majority of the downregulated genes in RVFV-infected samples compared to uninfected samples are not common between the liver and spleen. The biological processes in which these genes are involved depend on the specific functions of these organs (Fig. 5A to C). The majority of the genes downregulated in the liver upon RVFV infection code for transporters (*ABCA10*, *AQP8*, *RHBG*, and genes encoding proteins of the *SLC* solute carrier family) and hepatic enzymes. These hepatic enzymes are involved in diverse metabolic processes, including the metabolism of lipids (*ALDH1A1*, *CYP2E1*, *FADS1*, *NOTUM*, and *SCD*), steroid hormones (*CYP7A1*, *CYP8B1*, *DHCR7*, *HSD17B2*, and *TM7SF2*), amino acids (*CSAD*, *GAMT*, *GCAT*, and *GLYAT*), vitamins (*FMO1* and *PDXP*), carbohydrates (*PFKB1* and *TKFC*), and xenobiotics (*AOX1*, *CYP1A2*, and *GSTA1*) (Fig. 5A). Genes downregulated in the spleen after RVFV infection were mostly those encoding structural proteins (*ACTG2*, *MYOM1*, *TNXB*, and *TCHH*), enzymes (*ATP2A1*, *ALDH1L2*, and *ULK2*), and proteins with ligand binding activity (*HMCN2*, *LGALS12*, and *PAMR1*) (Fig. 5B). In both the liver and spleen tissues, the downregulation trend of the underexpressed genes was maintained during both phases of the infection (2 and 4 days post-infection).

### Host immune responses and metabolic pathways are markedly influenced by RVFV infection.

Besides inspecting the profiles of individual differentially expressed genes, we also performed a functional analysis of gene sets of interest based on the Gene Ontology (GO) database. Over-representation analysis (ORA) determines if known biological processes or molecular functions are significantly over-represented in a particular list of differentially expressed genes (36), while gene set enrichment analysis (GSEA) determines if an *a priori*-defined set of genes shows significantly coordinated differences (i.e., detection of small but consistent changes in the same direction) (37).

**FIG 6:**
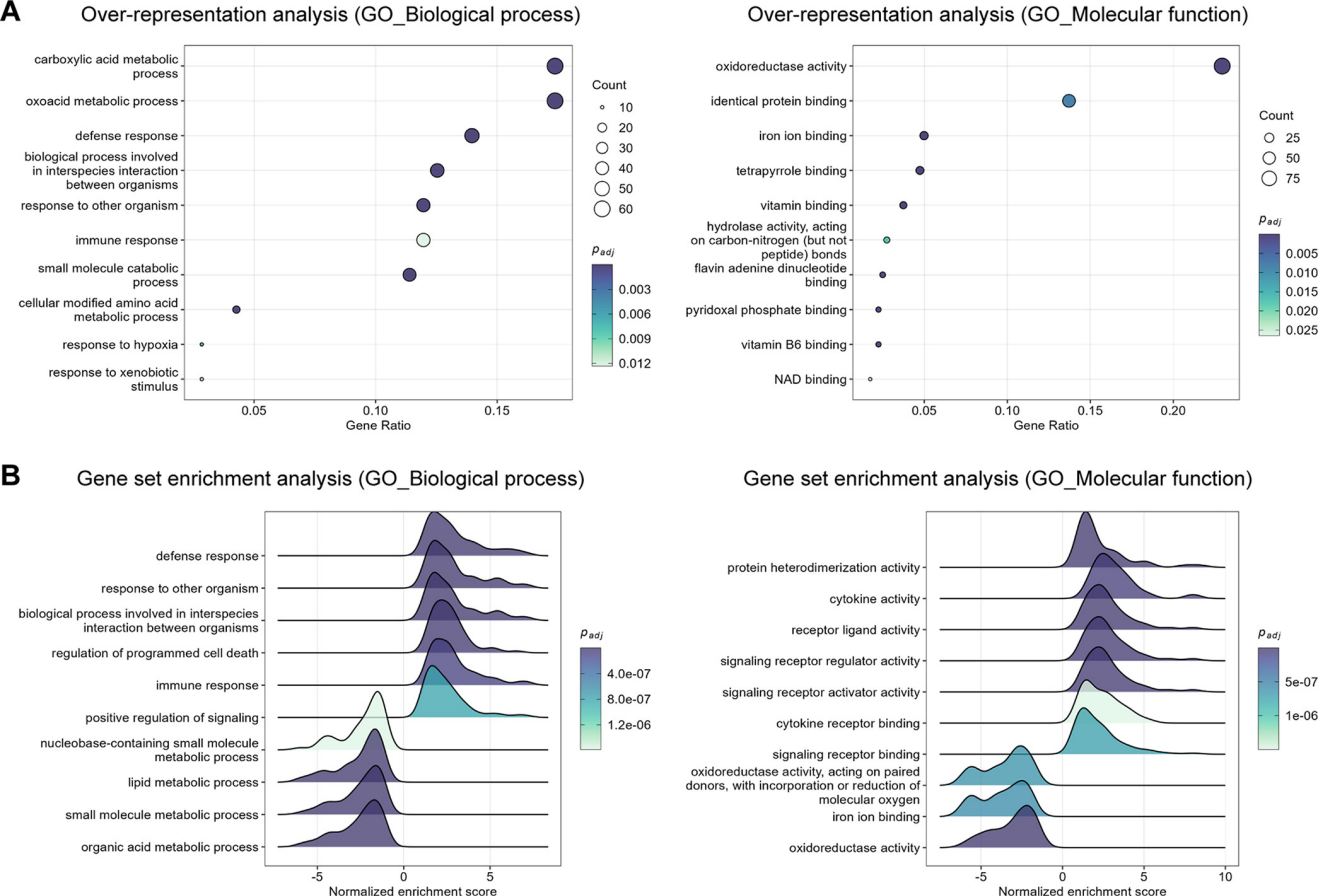
Top significantly enriched pathways altered in the liver in response to RVFV infection (2 days post-infection). (A) Gene Ontology (GO) biological process (left) and molecular function (right) over-representation analysis of genes differentially expressed in RVFV-infected liver tissue. The dot size represents the number of enriched genes associated with each GO term. Dots are color-coded according to their *P* values (adjusted by the Benjamini-Hochberg method). (B) GO biological process (left) and molecular function (right) gene set enrichment analysis in RVFV-infected liver tissue. Gene sets with positive normalized enrichment scores are upregulated, whereas gene sets with negative normalized enrichment scores are downregulated. Ridges are color-coded according to their *P* values (adjusted by the Benjamini-Hochberg method). The cutoff for significance for all the analyses was set to an adjusted *P* value <0.05. The top significantly enriched pathways altered in the liver in response to RVFV infection at 4 days post-infection are presented in Fig. S4 in the supplemental material.

Specifically for the liver, in terms of biological processes, ORA and GSEA showed significant enrichment for GO terms related to the immune response, positive regulation of signaling, regulation of programmed cell death, and the metabolism of diverse molecules (e.g., lipids, organic acids, and nucleobase-containing molecules) at the peak of the infection (2 days post-infection). Molecular functions associated with these processes included cytokine activity, cytokine receptor binding, binding to other ligands (e.g., iron and vitamins), and enzymatic activity (e.g., oxidoreductase and hydrolase) (Fig. 6A and B and Table S5). ORA and GSEA revealed very similar results for the 4 days post-infection time point (Fig. S4A and B and Table S5). Ridge plots in Fig. 6B depict that gene sets involved in the immune response were upregulated (positive normalized enrichment score), whereas gene sets involved in metabolic processes were downregulated (negative normalized enrichment score).

**FIG 7:**
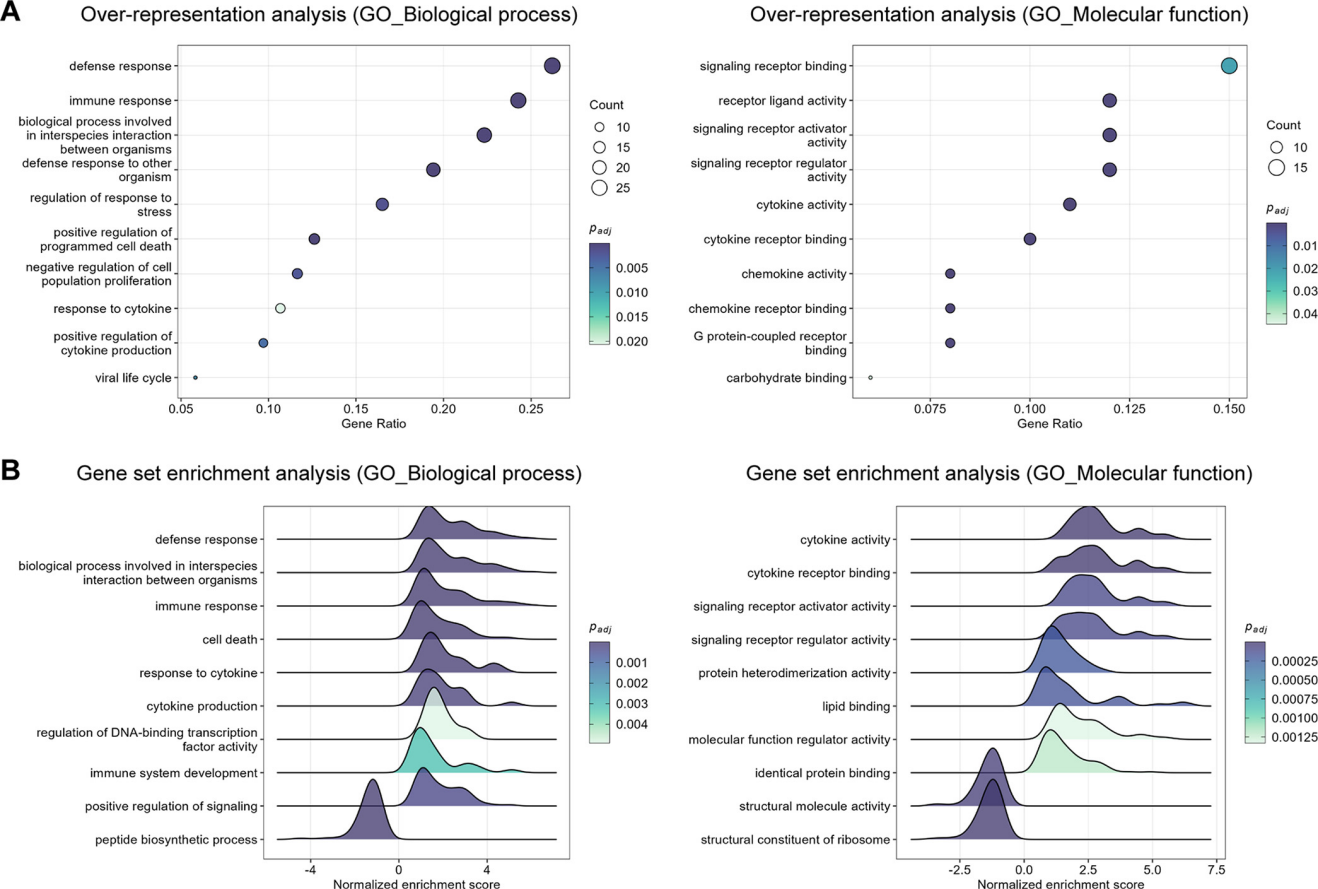
Top significantly enriched pathways altered in the spleen in response to RVFV infection (2 days post-infection). (A) Gene Ontology (GO) biological process (left) and molecular function (right) over-representation analysis of genes differentially expressed in RVFV-infected spleen tissue. The dot size represents the number of enriched genes associated with each GO term. Dots are color-coded according to their *P* values (adjusted by the Benjamini-Hochberg method). (B) GO biological process (left) and molecular function (right) gene set enrichment analysis in RVFV-infected spleen tissues. Gene sets with positive normalized enrichment scores are upregulated, whereas gene sets with negative normalized enrichment scores are downregulated. Ridges are color-coded according to their *P* values (adjusted by the Benjamini-Hochberg method). The cutoff for significance in all the analyses was set to an adjusted *P* value <0.05. The top significantly enriched gene sets altered in the spleen in response to RVFV infection at 4 days post-infection are presented in Fig. S5 in the supplemental material.

Similar to the liver, ORA and GSEA of the spleen data at the level of biological processes identified significant positive enrichment for GO terms involved in the immune response, regulation of the response to stress, regulation of programmed cell death, cytokine production, and the response to cytokines at 2 days post-infection. Regarding molecular functions, GO terms significantly enriched included cytokine activity, chemokine activity, signaling receptor binding, binding to other ligands (e.g., lipids and carbohydrates), and structural molecule activity (Fig. 7A and B and Table S5). Furthermore, GSEA identified GO terms related to the upregulation of apoptotic signaling, protein heterodimerization activity, translation regulator activity, and the downregulation of ion transmembrane transporter activity specifically at the 4 days post-infection time point (Fig. S5 and Table S5).

Additionally, ORA based on the Kyoto Encyclopedia of Genes and Genomes (KEGG) database corroborated the host’s most influenced pathways upon RVFV infection. Pathways related to the metabolism of diverse molecules (e.g., xenobiotics, fatty acids, retinol, and arachidonic acid), peroxisome proliferator-activated receptor (PPAR) signaling, and the biosynthesis of steroid hormones and cofactors were identified in the liver samples (Fig. S6A and Table S5). Pathways involved in viral infection, cytokine receptor interaction, NF-κB signaling, NOD-like receptor signaling, protein digestion, and apoptosis were identified in the spleen samples (Fig. S6B and Table S5).

### Levels of the host factor *LRP1* correlate with RVFV tissue tropism.

**FIG 8:**
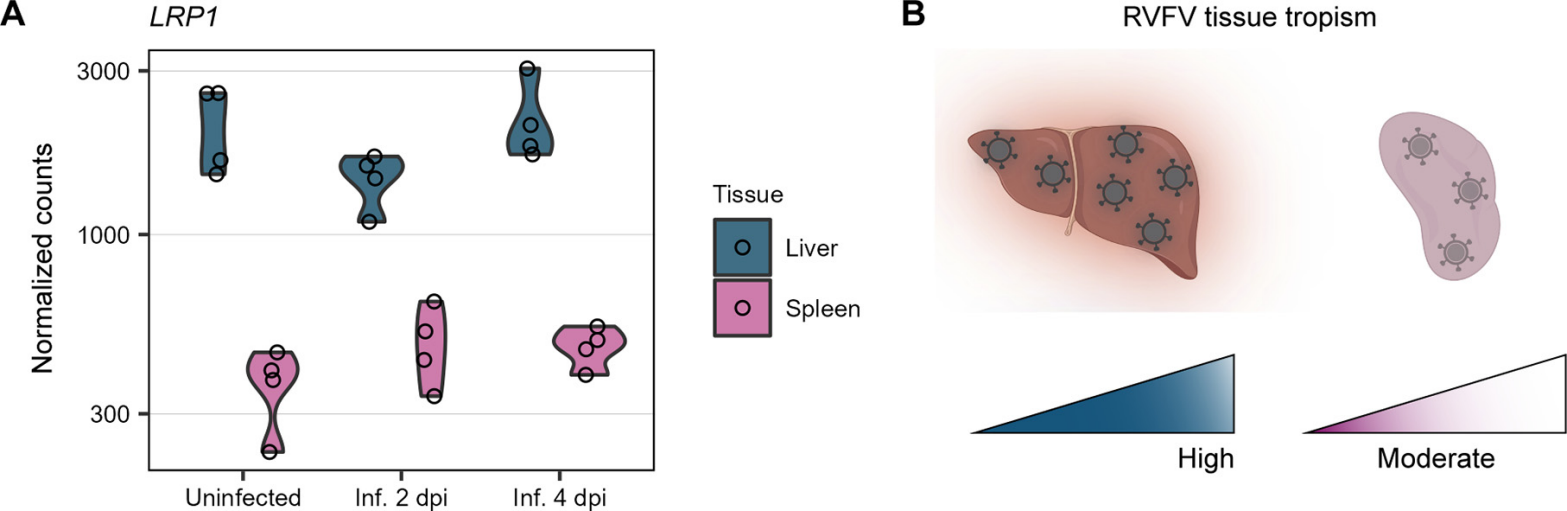
*LRP1* expression correlates with RVFV tissue tropism. (A) Expression profile of *LRP1* in the liver and spleen of lambs with different infection statuses. Dots represent individual normalized gene counts (on a log_10_ scale), and the shaded area shows the distribution of the samples within each group. (B) Schematic representation of RVFV tissue tropism. The elevated basal levels of *LRP1* in the liver correlated with the preference of RVFV for targeting this organ. Abbreviations: Inf., infected; dpi, days post-infection.

Recently, a genome-wide CRISPR screen identified low-density lipoprotein receptor-related protein 1 (LRP1) as a receptor for RVFV entry into host cells (38). We took advantage of our in-depth RNA-seq analysis to investigate *LRP1* expression. We observed that the levels of *LRP1* expression in lambs clearly differed between organs but were not affected by the infection status. *LRP1* was more abundantly expressed in the liver than in the spleen (Fig. 8A). Such high basal expression levels of the entry factor *LRP1* in the liver correlated with the preference of RVFV for targeting this organ (Fig. 8B).

## DISCUSSION

The acute onset of clinical signs and rapid progression to severe disease are features of RVFV infection (39). Experimental infections in rodents, ferrets, ruminants, and non-human primates have advanced our understanding of RVFV pathogenesis (22, 40–45). However, our knowledge of the underlying mechanisms is still rudimentary. Previous RNA-seq studies of *in vitro* infections and infections of non-target animals have unveiled important genes and pathways that are affected during RVFV infection (31–33). Nevertheless, RNA-seq studies with primary target animals and tissues have not yet been performed. Here, we examined the *in vivo* genome-wide transcriptional responses in target organs (liver and spleen) of RVFV-infected lambs. This work provides a broad view of the host factors affected by RVFV to better understand the molecular basis of RVFV pathogenesis.

Our study harnessed preserved tissue samples from a previous animal trial in which lambs were exposed to RVFV (34). Since this trial was not initially designed with the aim of performing a subsequent time course transcriptomic analysis, our study carries two inherent limitations. First, we did not have a conventional negative control group of uninfected lambs. To overcome this, we selected a specific set of samples from a group of lambs that were in theory exposed to the virus (through low-level exposure to mosquitoes) but that did not develop any signs of clinical disease and from which we could not detect infectious virus or viral RNA copies in the blood or the organs. In essence, these lambs had the same characteristics as those of lambs that have not been exposed to the virus. Arguably, these samples also represent a good control for the potential effects of mosquito bites.

As a second limitation, lambs from the two RVFV-infected groups were exposed to the virus via different routes. Lambs from group 2 were infected via intravenous injection, while lambs from group 3 were infected via the bites of infected mosquitoes. While it can be argued that different routes of infection may lead to distinct infection outcomes, we previously observed that RVFV infection of lambs via intravenous injection or mosquito bites leads to highly similar levels of infectious virus in the blood, comparable histopathological changes, and the same clinical outcomes (22). We are thus confident that these two above-mentioned limitations do not call into question the validity of our reported observations.

Previous studies by others on the host transcriptomic responses following RVFV infection reported the induction of defense responses (31–33). Here, we confirmed that upon infection, RVFV induced a strong activation of innate immune and inflammatory responses in both the liver and spleen tissues of lambs, principally through the sharp upregulation of IFN-induced antiviral host restriction factors and cytokine-mediated signaling pathways. The immune and inflammatory activation explains the typical high fever steadily observed through the critical 2- to 4-day-post-infection phase, reflecting the quick and sustained counteraction of the host for clearing the virus.

Among the top upregulated genes, *ISG20* and *ISG15* are of special interest. Indeed, an *in vitro* IFN-stimulated gene (ISG) expression screening identified potent antibunyaviral effects of ISG20 against a diverse panel of members of the *Peribunyaviridae*, *Hantaviridae*, and *Nairoviridae* families through its RNase activity (46). However, several members of the *Phenuiviridae* family (including RVFV) were described to resist the effects of ISG20 (46). We observed a significant upregulation of *ISG20* in infected tissues. This could mean either that RVFV may be susceptible to *ISG20*-mediated inhibition *in vivo* or that despite the high levels of *ISG20*, RVFV is indeed resistant to its antiviral effects (e.g., due to delayed kinetics of *ISG20* induction). *ISG15* has also been shown to exert antiviral activity against a wide range of viruses (47, 48), including the bunyavirus Crimean-Congo hemorrhagic fever virus (CCHFV) (family *Nairoviridae*) (49, 50). The over-expression of *ISG15* during RVFV infection likely reflects its antiviral role.

Several other top upregulated genes in both RVFV-infected liver and spleen tissues have also previously been implicated as key factors in the host antiviral response. For instance, *IFIT3* has been associated with the inhibition of adenovirus (51), rabies virus (52), Japanese encephalitis virus, herpesviruses (53), and even RVFV (31). *IFI6* was reported to block flavivirus replication (54), and *USP18* was reported to counteract infection by Sendai virus and encephalomyocarditis virus (55). Similar broad antiviral activities have been described for *XAF1* (56) and *LGALS3BP* (57). It is conceivable to think that the upregulation of these genes upon RVFV infection is the result of host cellular responses trying to limit viral replication. Thus, our results validate the role of commonly characterized antiviral host factors and expand the range of viruses against which they are known to exert restrictive activity.

The liver is the main target organ of RVFV, and infection generally leads to severe cellular damage that progresses to hepatic necrosis, often followed by inflammation with an infiltration of neutrophils (13, 39). Despite this tropism for the liver, we observed that only a small fraction of the tissue (approximately 10 to 20%) was infected in our samples. Most likely, RVFV-infected hepatocytes signal the viral threat to uninfected neighboring cells, inducing a strong antiviral state that counteracts the infection but compromises liver homeostasis for several days. Dozens of genes coding for enzymes involved in the metabolism of lipids, carbohydrates, proteins, nucleic acids, vitamins, and xenobiotics were markedly downregulated during peak viremia. In animals that recover from the infection, the generalized antiviral state, including transcriptional changes from uninfected neighboring cells, could explain the fast clearance of the virus. Once the levels of the virus decline, unaffected hepatocytes rapidly restore normal liver function.

The downregulation of oxidoreductases, hydrolases, transferases, and transporters, and overall liver failure are clear consequences of the observed tissue necrosis. Such a downregulation of hepatic enzymes responsible for essential metabolic processes possibly explains, for example, the icterus sometimes observed in RVFV-infected animals. Similarly, hemorrhage and coagulation disorders are likely to appear secondary to liver dysfunction due to the damaged liver’s incapacity to synthesize proteins crucial for blood homeostasis.

Interestingly, previous *in vitro* RNA-seq studies investigating the mammalian host response upon RVFV infection did not decidedly identify the downregulation of many important metabolic enzymes (31, 32). This apparent discrepancy is most probably explained by the fact that those studies employed small airway epithelial cells and embryonic kidney cells instead of more biologically relevant cells. One additional difference relates to the expression levels of genes involved in ion transport. Two solute carriers (*SLC24A2* and *SLC1A3*) were reported to be among the top upregulated genes upon RVFV infection (31), whereas we consistently found that several transporters from the same family (e.g., *SLC7A9*, *SLC22A1*, and *SLC22A9*) were significantly downregulated.

The spleen is a secondary target organ of RVFV. Necrosis of the spleen has been described as a common characteristic in naturally infected young lambs, often noticeable in both the red and white pulp (13). Although less evident than in the liver, the downregulation of genes coding for cellular structural proteins and genes involved in metabolic processes also denotes that the function of the spleen was seriously compromised as a consequence of RVFV infection. Importantly, it might be that the transcriptomic changes observed in the spleen are a combination of direct effects on infected splenocytes and affected immune cells that trafficked to the spleen.

LRP1, a newly identified host entry factor for RVFV (38), is ubiquitously expressed in numerous human tissues, but its expression levels are higher in the liver, placenta, brain, adipose tissue, and fibroblasts (data available from https://www.proteinatlas.org/ENSG00000123384-LRP1) (58). High expression levels of the LRP1 protein in these tissues can be associated with its natural function as a key molecule in intracellular lipoprotein metabolism (e.g., central cholesterol metabolism in the liver and progesterone biosynthesis in the placenta). In line with the human tissue-specific gene expression data, we found that the levels of *LRP1* expression in lambs were much higher in the liver than in the spleen. The high basal expression level of *LRP1* in the liver possibly explains, at least partially, the marked tropism of RVFV for human and ovine hepatocytes. RVFV also displays preferential tropism for placental cells in pregnant ewes (59), which normally leads to abortion storms during outbreaks (14). Whether there also is elevated basal *LRP1* expression in the sheep placenta and whether this favors placental infection remain to be investigated. Confirmation of a good correlation between *LRP1* gene expression and its protein levels in lambs awaits further investigation as well.

In summary, the transcriptomic analysis of RVFV-infected liver and spleen tissues of lambs presented here revealed the most important genes and pathways that mediate the strong innate immune response upon infection and the drastic decrease in tissue-specific function. Our results validated that IFN-mediated signaling pathways are key regulators of RVFV infection. Moreover, we uncovered the severely damaged liver metabolic function underlying the acute hepatitis and necrosis induced by RVFV. In addition, we highlighted the elevated basal expression level of *LRP1* in liver cells as a potential factor influencing RVFV tissue tropism. Altogether, the results of this study shed light on the extensive perturbance of the *in vivo* host transcriptome during critical phases of RVFV infection and provide new insights to better comprehend RVFV pathogenesis at the molecular level. Future studies could expand the current knowledge by concomitantly quantifying host and viral gene expression in individual cells. Transcriptomic profiling at single-cell resolution would make it possible to discriminate the innate immune responses of RVFV-infected cells from those of uninfected neighboring cells, ultimately improving our understanding of the molecular mechanisms employed by RVFV to overcome the host response.

## MATERIALS AND METHODS

### Tissue samples.

Liver and spleen samples were selected from another study aimed at comparing RVFV infection in Texel-Swifter lambs after low-exposure (3 mosquitoes) and high-exposure (28 to 31 mosquitoes) challenges (34). Mosquitoes used for the challenge were initially fed on lambs 2 days after the intravenous injection of 10^5^ median tissue culture infectious doses (TCID_50_) of RVFV strain 35/74 (60). For the purpose of the present study, 4 samples per group were selected from (i) non-responsive lambs (that did not develop viremia or disease) necropsied 8 days after low-level mosquito exposure (group 1), (ii) lambs presenting with high viremia necropsied 2 days after intravenous injection (group 2), and (iii) lambs presenting with high viremia necropsied 4 days after high-level mosquito exposure (group 3). A summary of the different groups is presented in Table 1. The group numbers and descriptions presented in Table 1 belong exclusively to the present study, but to enable traceability, the original identification numbers of the selected samples are included. Within each group, samples were selected based on similar profiles of infectious titers and viral RNA copy numbers in the blood, liver, and spleen. The infectious titers and viral RNA copy numbers were determined by an endpoint dilution virus isolation assay and M-segment-specific quantitative reverse transcription PCR (RT-qPCR), respectively, as described previously (34). Liver and spleen samples were long-term preserved in RNA*later* (Invitrogen) at ≤ −65°C.

**TABLE 1:** Description, route of exposure, identification number, sex, and age of the lambs from a previous animal study^*a*^ selected for transcriptomic analysis

Group	Description^*b*^	Route of exposure	Animal ID	Sex	Age (at challenge) (wks)
1	Uninfected	Mosquito bite (low exposure)	276	Female	10
			277	Male	
			278	Female	
			279	Female	

2	Infected 2 dpi	Intravenous injection	271	Female	10
			272	Female	
			273	Male	
			274	Female	

3	Infected 4 dpi	Mosquito bite (high exposure)	4618	Male	10
			4619	Female	
			4624	Male	
			4642	Female	

a See reference 34.

b dpi, days post-infection.

### Histology and immunohistochemistry.

Tissue samples were fixed in 10% neutral buffered formalin for 48 h and processed routinely into paraffin blocks. Sections were cut on silane-coated glass slides and dried in a 37°C incubator for at least 48 h. After deparaffinization in xylene and rehydration in a series of graded alcohols, sections were stained routinely with hematoxylin and eosin (H&E) or immunostained for RVFV antigen. For immunostaining, heat-induced epitope retrieval was applied by autoclaving the sections for 15 min at 121°C in citrate buffer (pH 6) (Vector Laboratories). Monoclonal antibody 4-D4 (61), directed against the Gn glycoprotein, was used as the primary antibody. Goat polyclonal anti-mouse horseradish peroxidase (HRP)-conjugated IgG (Invitrogen) was used as the secondary antibody, with diaminobenzidine (DAB^+^) (Dako, Agilent) as the substrate. Sections were counterstained with Mayer’s hematoxylin and mounted permanently. Images were taken with an Olympus BX51 microscope equipped with a high-resolution digital camera.

### RNA isolation.

Following thawing, liver and spleen tissue fragments of approximately 80 to 260 mg were washed once in 500 μL of Dulbecco’s phosphate-buffered saline (DPBS) (Gibco) to remove excess RNA*later* and then placed into Lysing Matrix D tubes (MP Biomedicals) with 1 mL of TRIzol (Invitrogen). Tissue fragments were homogenized with at least 2 cycles in a FastPrep-24 homogenizer (MP Biomedicals) at 6 m/s for 50 s each cycle until complete homogenization was achieved. When required, to improve the homogenization of complex samples, 2 glass beads per tube were added. Homogenates were centrifuged at 20,800 relative centrifugal force (rcf) (Eppendorf 5417R) for 40 min at 4°C, and the cleared supernatants were kept. Total RNA extractions of 350 μL of lysed samples were performed with the Direct-zol RNA MiniPrep kit (Zymo Research), according to the manufacturer’s instructions, with an additional centrifugation step after the addition of RNA wash buffer. Lysed preparations were treated with 30 U of DNase I for 15 min. Total RNA was eventually eluted in 30 μL of DNase/RNase-free water. The RNA concentration and purity were measured on a NanoDrop One instrument (Thermo Fisher Scientific), and samples were stored at ≤ −65°C until further use.

### RNA-seq.

Isolated RNA was sent for next-generation sequencing to GenomeScan B.V. (Leiden, The Netherlands). The RNA integrity was determined using a Fragment Analyzer system (Agilent) or a 2100 Bioanalyzer (Agilent). Samples were prepared using the NEBNext Ultra II Directional RNA Library Prep Kit for Illumina (catalog number E7760S/L; New England BioLabs [NEB]) according to the instructions of the manufacturer. Briefly, host poly(A) mRNA was isolated from total RNA using oligo(dT) magnetic beads. After the fragmentation of the mRNA, cDNA was synthesized, sequencing adapters were ligated, and the fragments were PCR amplified. The quality and yield of the sequencing library were determined using a Fragment Analyzer (Agilent). The resulting products had a size distribution with a broad peak between 300 and 500 bp. Clustering and sequencing of 1.1 nM DNA samples were performed using an Illumina NovaSeq 6000 instrument with NovaSeq control software NCS version 1.7, according to the instructions of the manufacturer. The sequence length of the short reads ranged between 151 and 159 bp.

### RNA-seq data preprocessing.

Raw sequencing reads were processed according to the nf-core/RNA-seq pipeline (62) version 3.4 (https://doi.org/10.5281/zenodo.1400710). Quality control was performed with FastQC (63) version 0.11.9 and MultiQC (64) version 1.10.1. Based on the high mean sequence quality (average Phred score >30) (see Fig. S1 in the supplemental material), only soft trimming was required. Adapter sequences were removed with Cutadapt (65) version 3.4. Trimmed reads had an average length ranging between 131 and 150 bp. Reads were deduplicated based on unique molecular identifiers (UMIs) using UMI-tools (66) version 1.1.2. Unique reads were aligned to the sheep (Ovis aries) NCBI reference genome version 4.0 (Oar_v4.0) (https://www.ncbi.nlm.nih.gov/assembly/GCF_000298735.2/) with HISAT2 (67) version 2.2.0 and quantified using featureCounts (68) (within the *Rsubread* package version 2.4.3). Unannotated genes were automatically assigned a *LOC* prefix gene identification nomenclature designation based on genomic position. However, the corresponding gene name, when available, was manually assigned for the plots presented in Fig. 4A to C and Fig. 5A to C.

### Transcriptome analysis.

The data set was filtered to remove low-count genes (at least 2 samples should have counts of 3 or higher for a gene to be kept). Pairwise differential gene expression analysis was performed with DESeq2 (69) version 1.38.3 in R (70) version 4.2.2, using an absolute log_2_ fold change threshold of 1 and an alpha value of 0.05. DESeq2 models the count data with a negative binomial distribution using a generalized linear model. Wald test *P* values <0.05 (adjusted with the Benjamini-Hochberg method) were considered significant. For the initial exploration of the data, Euclidean distances between samples were calculated, and principal component analysis was conducted after applying a variance-stabilizing transformation to the data. For visualization and ranking purposes, the log_2_ fold change was shrunken using the adaptive shrinkage estimator from the *ashr* package (71) version 2.2-54. Heat maps were created with the R package *pheatmap* version 1.0.12 (https://CRAN.R-project.org/package=pheatmap) or *ComplexHeatmap* (72) version 2.14.0. Euler diagrams were plotted with eulerr (73) version 6.1.1. Volcano plots were generated using *EnhancedVolcano* (74) version 1.16.0.

Functional ORA and GSEA were performed with *clusterProfiler* (75, 76) version 4.6.0, based on the GO and KEGG databases. When required, the clusterProfiler function *simplify* was used to reduce the redundancy of enriched GO terms. *P* values <0.05 (adjusted by the Benjamini-Hochberg method) of a one-sided version of Fisher’s exact test were considered significant for ORA. *P* values <0.05 (adjusted by the Benjamini-Hochberg method) of a permutation test were considered significant for GSEA.

### Data analysis and visualization.

Prism 9 (GraphPad Software) was used to generate graphs of infectious titer and viral RNA copy number data. Transcriptomic data were analyzed and plotted in R (70) version 4.2.2, using the above-mentioned R packages. Statistical tests differed per analysis and are indicated in the description of each analysis and the corresponding figure legends. *P* values ≥0.05 were considered not significant.

### Ethics statement.

The animal experiment carried out within the scope of another study (34), from which organ samples were obtained for analysis, was conducted in accordance with European regulations (European Union directive 2010/63/EU) and the Dutch Law on Animal Experiments (Wod, identification number BWBR0003081). Permissions were granted by the Dutch Central Authority for Scientific Procedures on Animals (permit numbers AVD4010020185564 and AVD4010020187168). Specific procedures were approved by the Animal Ethics Committees of Wageningen Research.

### Data availability.

Raw sequencing data were deposited at the NCBI Sequence Read Archive (SRA) under BioProject accession number PRJNA935986.
