# Supplementary material for: Transcriptomic Profiling Reveals Intense Host-Pathogen Dispute Compromising Homeostasis during Acute Rift Valley Fever Virus Infection
Source: J Virol. 2023 Jun 12;97(6):e00415-23. doi: 10.1128/jvi.00415-23 (PMC10308945; doi:10.1128/jvi.00415-23)
Supplement: Supplemental file 1 — Fig. S1 to S6 Tables S1 and S2. Download jvi.00415-23-s0001.docx, DOCX file, 2.1 MB [file jvi.00415-23-s0001.docx]

**Supplementary Information**

**Transcriptomic profiling reveals intense host-pathogen dispute compromising homeostasis during acute Rift Valley fever virus infection**

Erick Bermúdez-Méndez, Paolo Angelino, Lucien van Keulen, Sandra van de Water, Barry Rockx, Gorben P. Pijlman, Angela Ciuffi, Jeroen Kortekaas, Paul J. Wichgers Schreur*

* Correspondence: paul.wichgersschreur@wur.nl


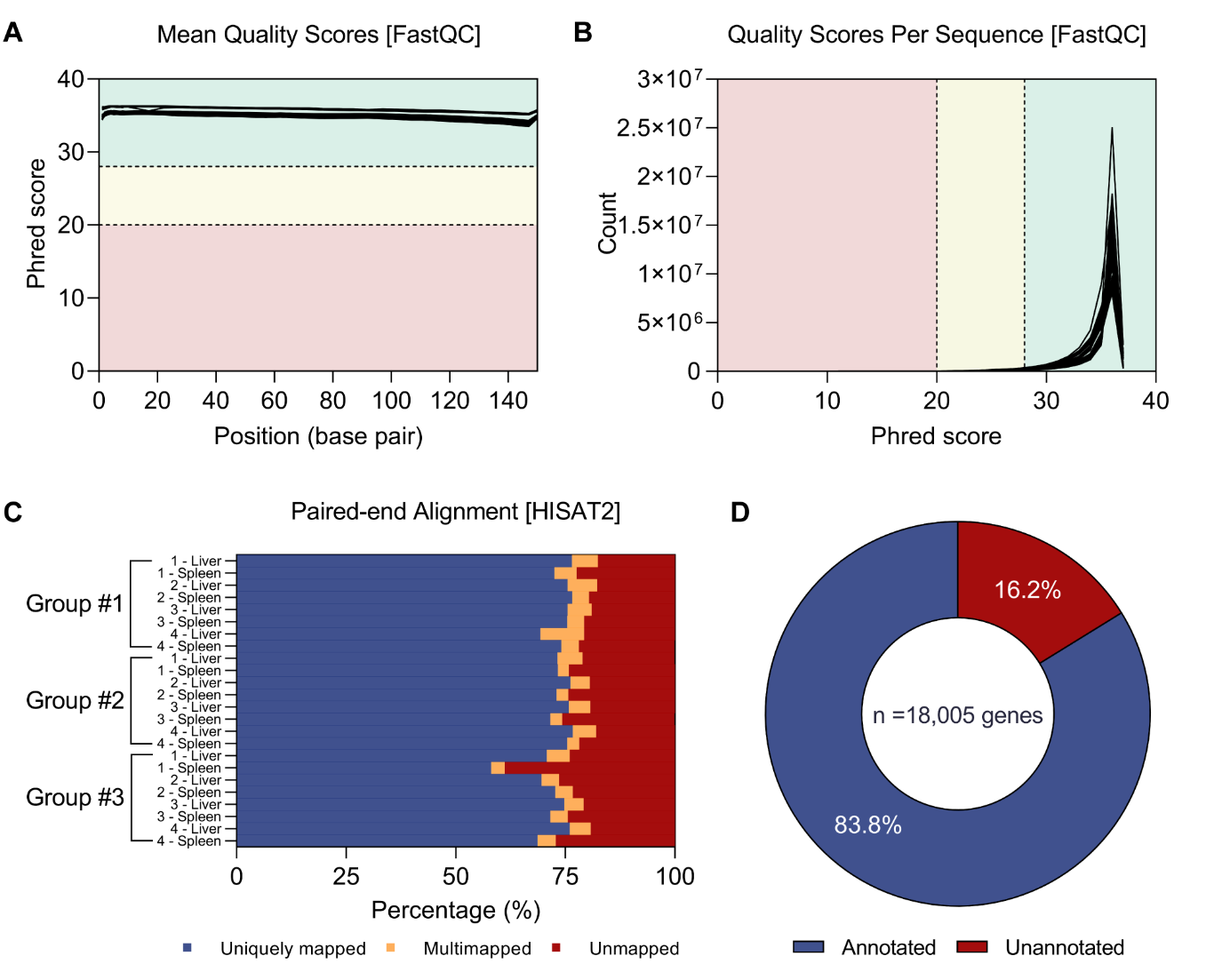


**Supplementary Fig. 1 RNA-seq raw data quality control and pre-processing. A** Mean quality (Phred) scores across each position in the raw reads. **B**Histogram of per sequence quality (Phred) scores for the raw reads. Raw data quality control was performed with FastQC^1^. Individual lines represent the scores for each of the 48 raw reads. Dashed lines divide the plotting area into three regions according to the quality scores. Red indicates low quality (Phred score < 20), yellow indicates middle quality (20 ≤ Phred score < 28) and green indicates high quality (Phred score ≥ 28). **C** Alignment of unique reads to the sheep (*Ovis aries*) NCBI reference genome using HISAT2^2^. Single mate alignment counts were halved to tally with pair counts properly. **D** Fraction of input genes for analysis annotated in the reference genome.


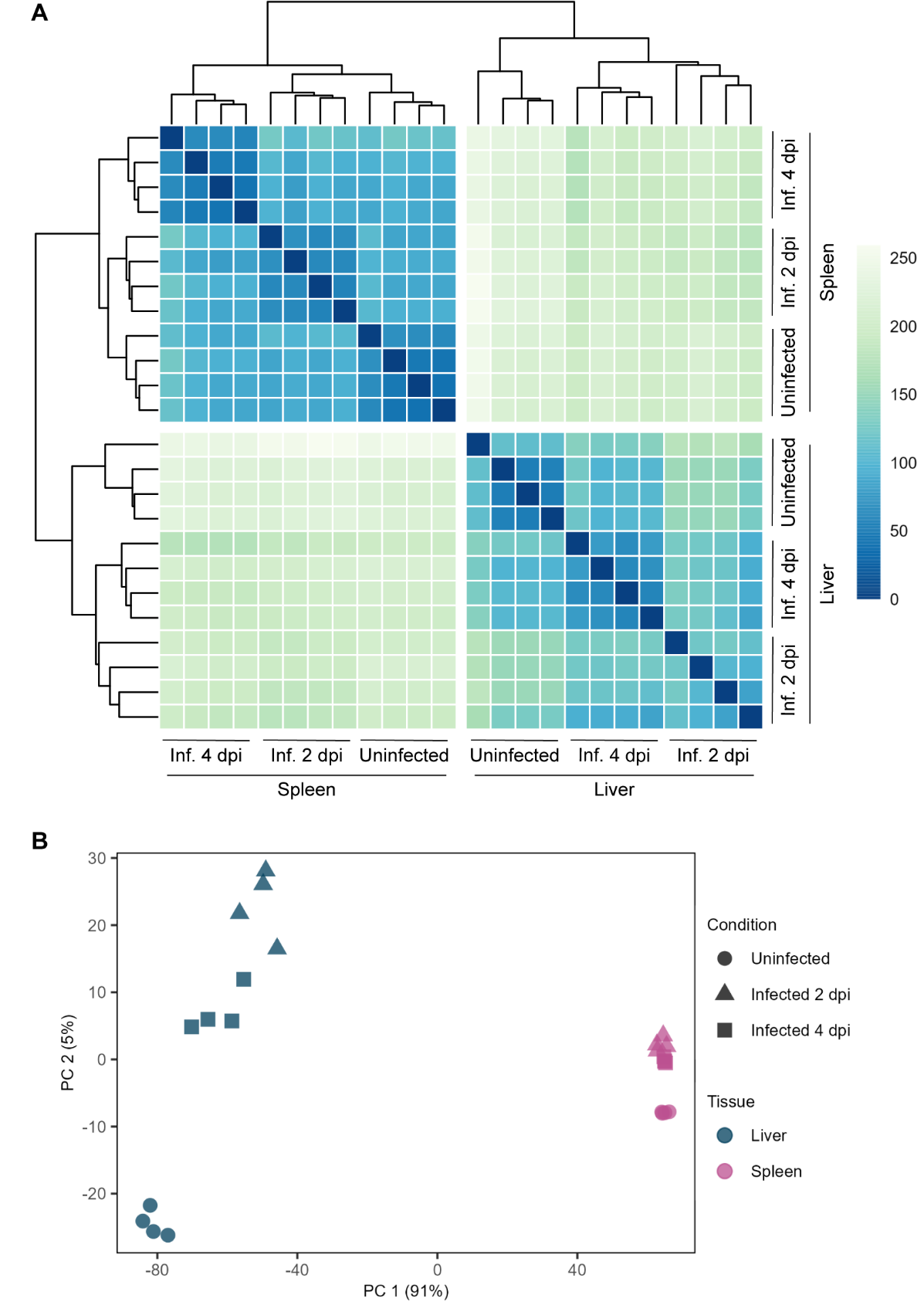


**Supplementary Fig. 2 Distinct tissue-specific gene expression profiles. A** Heat map of euclidean distances between all the liver and spleen tissue samples. Based on the calculated distance, each cell is color-coded in shades of a sequential gradient ranging from dark blue (close distance, implying similarity) to light green (far distance, implying dissimilarity). **B** Principal component analysis of all the liver and spleen tissue samples. The sharp separation of the samples into two well-defined clusters indicates that gene expression profiles clearly differ between tissue types. Abbreviations: Inf., infected; dpi, days post-infection.


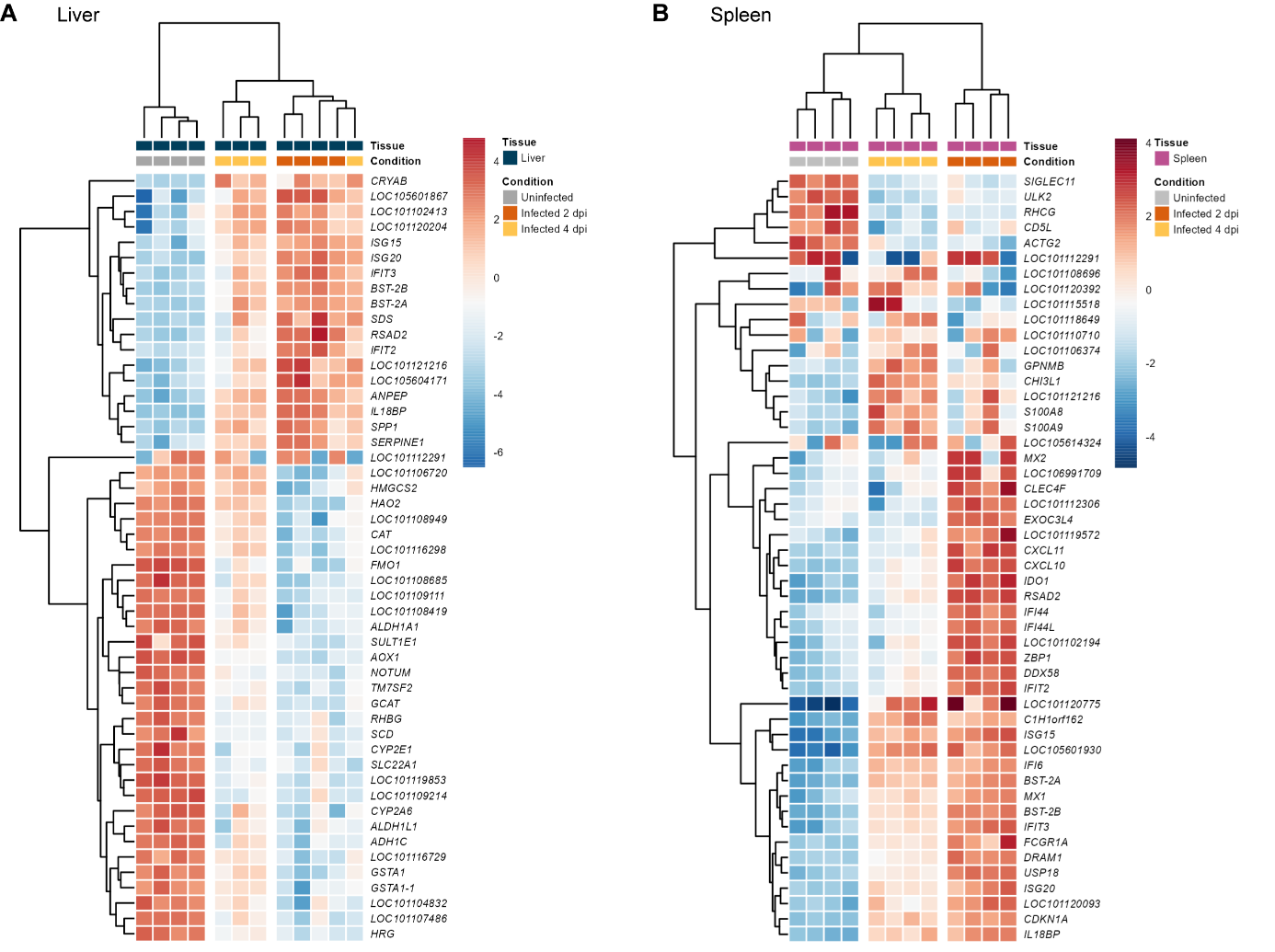


**Supplementary Fig. 3 Top variable genes differentially expressed upon RVFV infection. A, B** Heat maps of the top 50 most variably expressed genes after RVFV infection in liver (**A**) and spleen (**B**) tissues. Based on the gene expression patterns of the most variably expressed genes, samples were hierarchically clustered into three groups. To represent the magnitude of the log_2_ fold change of each gene compared to the mean gene expression, cells are color‑coded in shades of a gradient ranging from dark blue (low) to dark red (high). Abbreviation: dpi, days post-infection.


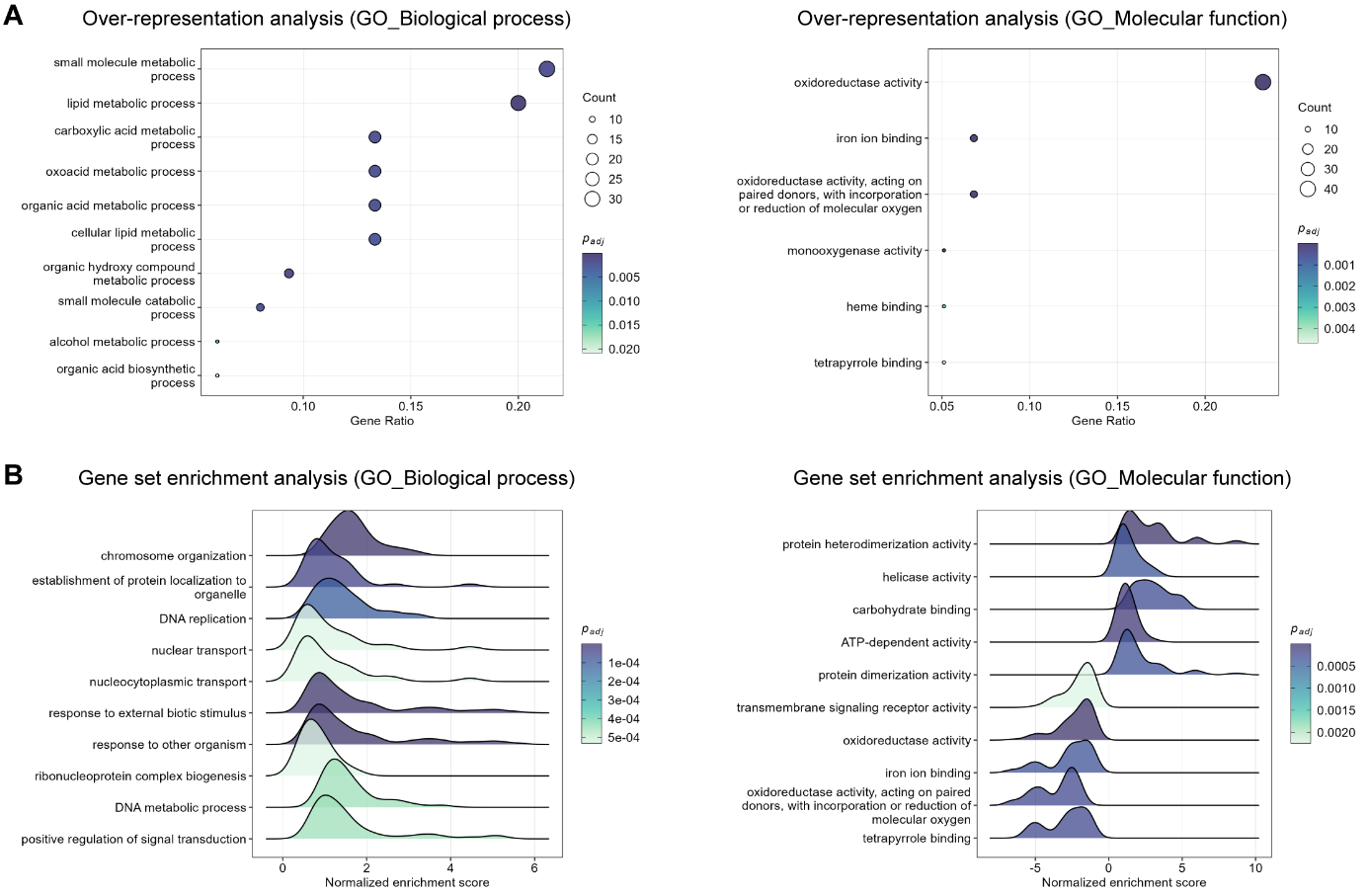


**Supplementary Fig. 4 Top significantly enriched pathways altered in the liver in response to RVFV infection (4 days post-infection). A**Gene ontology (GO) biological process (left) and molecular function (right) over‑representation analysis of genes differentially expressed in RVFV-infected liver tissue. Dot size represents the number of enriched genes associated to each GO term. Dots are color-coded according to their adjusted *p*value (Benjamini‑Hochberg method). **B** GO biological process (left) and molecular function (right) gene set enrichment analysis in RVFV-infected liver tissue. Gene sets with positive normalized enrichment scores are upregulated, whereas gene sets with negative normalized enrichment scores are downregulated. Ridges are color‑coded according to their adjusted *p*value (Benjamini‑Hochberg method). Cut-off for significance in all the analyses was set to a *p* adjusted value < 0.05.


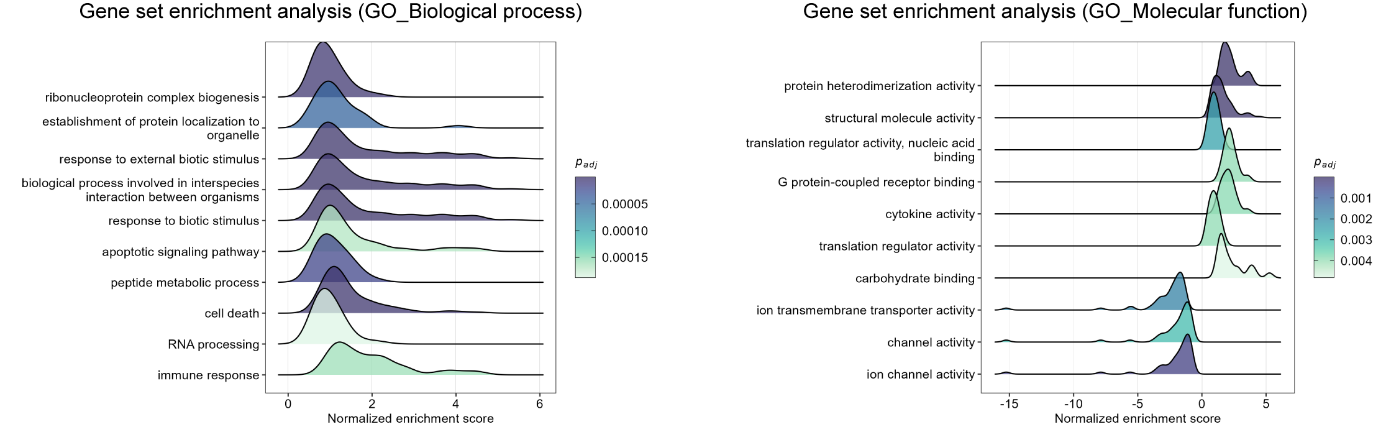


**Supplementary Fig. 5 Top significantly enriched gene sets altered in the spleen in response to RVFV infection (4 days post-infection).** Gene ontology (GO) biological process (left) and molecular function (right) gene set enrichment analysis in RVFV-infected spleen tissue. Gene sets with positive normalized enrichment scores are upregulated, whereas gene sets with negative normalized enrichment scores are downregulated. Ridges are color-coded according to their adjusted *p*value (Benjamini‑Hochberg method). Cut-off for significance in all the analyses was set to a *p* adjusted value < 0.05.


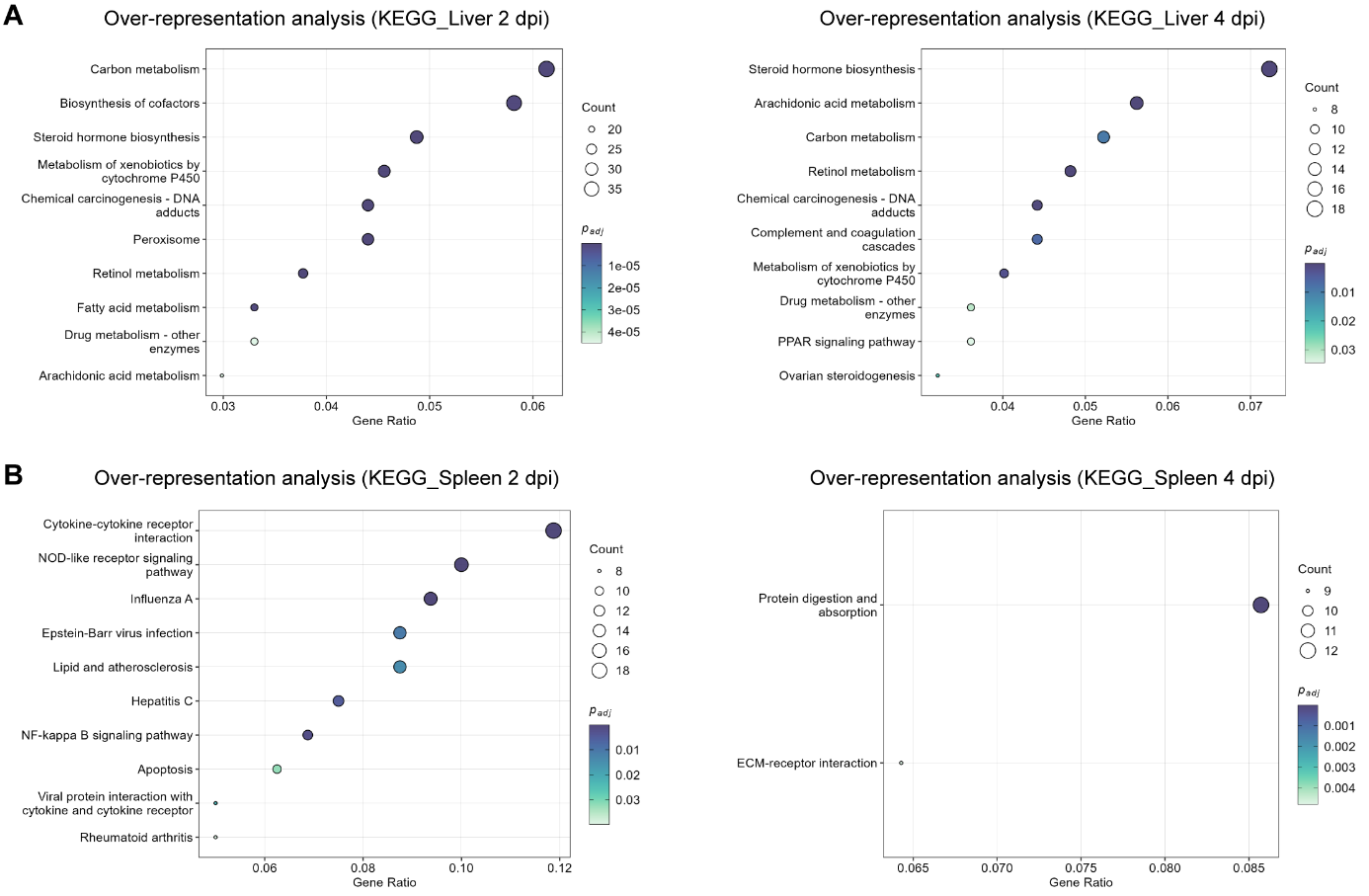


**Supplementary Fig. 6 Top significantly enriched KEGG pathways altered in the liver and spleen in response to RVFV infection. A, B**Kyoto Encyclopedia of Genes and Genomes (KEGG) pathways over‑representation analysis of genes differentially expressed in RVFV-infected liver (**A**) and spleen (**B**) tissues at 2 (left) and 4 (right) days post-infection. Dot size represents the number of enriched genes associated to each KEGG pathway. Dots are color‑coded according to their adjusted *p*value (Benjamini‑Hochberg method). Cut-off for significance in all the analyses was set to a *p* adjusted value < 0.05. Abbreviation: dpi, days post-infection.

**Supplementary Table 1** Quality of isolated RNA used for next-generation sequencing.

| Group | Sample ID | RNA quality number (RQN)* |
| --- | --- | --- |
| Group #1 | 1 - Liver | 7.1 |
|  | 1 - Spleen | 6.4 |
|  | 2 - Liver | 6.3 |
|  | 2 - Spleen | 6.2 (RIN) |
|  | 3 - Liver | 6.1 |
|  | 3 - Spleen | 7.2 |
|  | 4 - Liver | 4.1 (RIN) |
|  | 4 - Spleen | 6.3 (RIN) |
| Group #2 | 1 - Liver | 5.2 (RIN) |
|  | 1 - Spleen | 5.2 |
|  | 2 - Liver | 6.8 |
|  | 2 - Spleen | 7.3 |
|  | 3 - Liver | 6.3 |
|  | 3 - Spleen | 7.3 |
|  | 4 - Liver | 6.3 |
|  | 4 - Spleen | 7.0 |
| Group #3 | 1 - Liver | 6.2 |
|  | 1 - Spleen | 7.2 |
|  | 2 - Liver | 6.5 |
|  | 2 - Spleen | 7.5 |
|  | 3 - Liver | 7.3 |
|  | 3 - Spleen | 7.8 |
|  | 4 - Liver | 6.6 |
|  | 4 - Spleen | 7.1 |

* For some samples, the RNA integrity number (RIN) is reported instead of the RQN.

**Supplementary Table 2** Summary of the next-generation sequencing run.

| Group | Sample ID | Total reads (M)  [FastQC] | Average Phred score  [FastQC] | % Aligned  [HISAT2] |
| --- | --- | --- | --- | --- |
| Group #1 | 1 - Liver | 21.7 | 35.4 | 82.4 |
|  | 1 - Spleen | 24.6 | 35.1 | 77.6 |
|  | 2 - Liver | 26.6 | 35.4 | 82.3 |
|  | 2 - Spleen | 23.3 | 35.3 | 80.4 |
|  | 3 - Liver | 24.1 | 35.3 | 81.0 |
|  | 3 - Spleen | 22.4 | 35.4 | 79.3 |
|  | 4 - Liver | 20.9 | 35.3 | 79.3 |
|  | 4 - Spleen | 25.7 | 35.5 | 78.2 |
| Group #2 | 1 - Liver | 28.6 | 35.2 | 79.0 |
|  | 1 - Spleen | 28.8 | 35.3 | 75.8 |
|  | 2 - Liver | 20.0 | 35.3 | 80.6 |
|  | 2 - Spleen | 21.8 | 35.3 | 75.7 |
|  | 3 - Liver | 27.9 | 35.3 | 80.7 |
|  | 3 - Spleen | 20.5 | 35.2 | 74.3 |
|  | 4 - Liver | 24.6 | 35.4 | 82.0 |
|  | 4 - Spleen | 38.7 | 35.5 | 78.2 |
| Group #3 | 1 - Liver | 23.2 | 35.1 | 76.0 |
|  | 1 - Spleen | 24.1 | 34.1 | 61.1 |
|  | 2 - Liver | 21.4 | 35.3 | 73.6 |
|  | 2 - Spleen | 25.2 | 35.5 | 76.7 |
|  | 3 - Liver | 22.5 | 35.3 | 79.2 |
|  | 3 - Spleen | 25.6 | 35.4 | 75.6 |
|  | 4 - Liver | 26.7 | 35.3 | 80.8 |
|  | 4 - Spleen | 25.8 | 35.1 | 72.8 |

In the column headings, the bioinformatic tool from which the information was extracted is specified within square brackets.

**Supplementary References**

1. Andrews, S. (2010). FastQC: a quality control tool for high throughput sequence data. Available online at: <http://www.bioinformatics.babraham.ac.uk/projects/fastqc/>.

2. Kim, D., Paggi, J.M., Park, C., Bennett, C., and Salzberg, S.L. (2019). Graph-based genome alignment and genotyping with HISAT2 and HISAT-genotype. Nat Biotechnol *37*, 907–915. 10.1038/s41587-019-0201-4.
